# A Pilot Approach Investigating the Potential of Crop Rotation With Sainfoin to Reduce *Meloidogyne enterolobii* Infection of Maize Under Greenhouse Conditions

**DOI:** 10.3389/fpls.2021.659322

**Published:** 2021-04-16

**Authors:** Milad Rashidifard, Samad Ashrafi, Sarina Claassens, Torsten Thünen, Hendrika Fourie

**Affiliations:** ^1^Unit for Environmental Sciences and Management, North-West University, Potchefstroom, South Africa; ^2^Julius Kühn-Institut, Federal Research Centre for Cultivated Plants, Institute for Epidemiology and Pathogen Diagnostics, Braunschweig, Germany; ^3^Julius Kühn-Institut, Federal Research Centre for Cultivated Plants, Institute for Crop and Soil Science, Braunschweig, Germany

**Keywords:** crop rotation, maize, management, *Meloidogyne*, *Onobrychis viciifolia*, plant-parasitic nematodes, sainfoin

## Abstract

Root-knot nematodes (RKNs) are one of the most important plant-parasitic nematodes of cereal crops in sub-Saharan Africa. This study was designed to evaluate the rotation effects of different cultivars of sainfoin (Esparsette, Perly, Taja and Visnovsky), soybean (DM-5953-RSF) and alfalfa (BAR 7) with maize (P-2432-R), on a *Meloidogyne enterolobii* population, compared to monoculture maize. The results showed that sainfoin (Perly and Esparsette) and alfalfa had significantly (*P* ≤ 0.05) lower numbers of *M. enterolobii* eggs and second stage juveniles (J2) compared to the monoculture maize in the first experiment. However, in the repeat experiment all treatments had significantly (*P* ≤ 0.05) lower numbers of eggs and J2 compared to monoculture maize. Rotation of sainfoin Esparsette/maize resulted in the lowest numbers of eggs and J2 (91 and 202, respectively) in the first and repeat experiments. Rotation of sainfoin Esparsette/maize reduced *M. enterolobii* population density by 81 and 60% in the first and repeat experiments, respectively, followed by alfalfa (54 and 43%, respectively). Ultimately, substantial variation was evident in terms of the efficacy of different sainfoin cultivars with regards to their effect on nematode reduction when used in rotation with maize.

## Introduction

Due to an increasing human population, more food has to be produced with cereal crops representing the staple food source for human and livestock consumption worldwide (FAO, 2017). Production of cereal crops in Africa, however, declined by 4.6% in 2019 in comparison to 2018 (FAO, 2020) and is a risk to food security in Africa. In South Africa, maize (*Zea mays*) is the most important cereal crop, but production of the crop is adversely affected due to various diseases and pests. Among others, plant-parasitic nematodes (PPNs) represent one of the major limiting biotic factors in maize cropping systems (Fourie et al., 2017; Mc Donald et al., 2017). The most economically important PPNs in maize production areas in South Africa are root-knot nematodes (*Meloidogyne* spp.) and root lesion nematodes (*Pratylenchus* spp.) (Degenkolb and Vilcinskas, 2016; Mc Donald et al., 2017). High population densities of *Meloidogyne* and/or *Pratylenchus* spp. have been reported recently for maize and other rotation crops (Fourie et al., 2017; Mc Donald et al., 2017). Earlier studies demonstrated that root-knot nematodes parasitism caused up to 60% yield losses to South African maize crops (Riekert and Henshaw, 1998). While *M. incognita* and *M. javanica*, followed by *M. arenaria*, are considered the predominant root-knot nematode species parasitizing local maize crops, *M. enterolobii* has also been discovered in a major maize producing area of the country (Pretorius, 2018). The latter species is known for its higher pathogenicity toward agricultural crops and especially for its virulence since it overcomes resistance in crops that are effective to its counterpart thermophilic species (Kiewnick et al., 2009).

To minimize damages caused by PPNs and increase crop production, combating destructive nematode pests should be considered a priority. Use of chemical nematicides was one of the most effective approaches in nematode management in the last decades (Fernández et al., 2001). Nevertheless, increasing awareness of the toxicity of pesticides (to animals, humans and the environment) and the emergence of resistance against synthetic nematicides (Kishi et al., 1995; Kaplan, 2004) are some of the reasons for the use of more environmentally friendly nematode management strategies. Therefore, to maintain PPN populations under a certain threshold, biological and cultural management strategies would play a major role in the future. In this context, the well-planned use of crop rotation is one of the possible management practices that can reduce the PPN populations in maize-based agriculture systems. However, most of the crops, especially legumes, which are used in rotation with maize in sub-Saharan Africa (SSA) and other parts of the world are parasitized by the same nematode pests of maize (Fourie et al., 2017; Coyne et al., 2018; Dababat and Fourie, 2018). Ultimately, it is crucial to find suitable crops that can be used as a rotation or cover crop in maize-based cropping systems, and that can suppress PPN densities. Sainfoin (*Onobrychis viciifolia*) can be an appropriate alternative, particularly due to its chemical properties, which amongst others include the production of condensed tannins with anthelmintic properties (Novobilský et al., 2013). Sainfoin was used for animal feeding in the past but after the green revolution it was replaced by alfalfa (*Medicago sativa*) especially because of alfalfa’s potential to produce higher yields. The yield range of sainfoin is 20–30% lower than that of alfalfa in the long-term, however, in the first cut, sainfoin yields are higher than that of alfalfa (Baldridge and Lohmiller, 1990). Sainfoin is a perennial, non-bloating forage legume, which grows up to 20–90 cm tall. It is highly palatable as a fodder source to ruminants when compared to alfalfa (Hume and Withers, 1985). Sainfoin is tolerant to drought and harsh environmental conditions due to the large and deep root system. A major trait, especially in regard to the topic of this study is that this plant is not subjected to major pests of alfalfa (Kaldy et al., 1979; Lance, 1980). The nematicidal activity of condensed tannins of sainfoin on the gastrointestinal parasitic nematodes of ruminants has been well documented (Novobilský et al., 2013; Mueller-Harvey et al., 2019). Consumption of low or moderate concentrations of condensed tannins is also associated with positive effects e.g., the increase in milk, growth and wool production in herbivore animals (Hoste et al., 2006) and a decrease in greenhouse gas emissions (Mueller-Harvey, 2006).

Both root-knot (*Meloidogyne* spp.) and stem nematodes (*Ditylenchus dipsaci*) have been reported to parasitize sainfoin in the US (Mathre, 1968). Most sainfoin cultivars used in the US, for example, showed high susceptibility when evaluated against *M. hapla* populations (Wofford and Gray, 1987; Wofford et al., 1989). By contrast, another study showed tolerance of sainfoin “Shoshone” to *M. hapla* (Gray et al., 2006b). In the last decades, research on sainfoin from a plant nematology point of view has ceased. Currently no information about the effect of sainfoin on PPN populations when grown in rotation with maize is available. This research aimed to elucidate the effect of four different sainfoin cultivars to *Meloidogyne enterolobii* population densities when used in a glasshouse set-up in a maize-based rotation system and compared the nematode susceptibility of one maize, one soybean (*Glycine max*) (a common rotation crop for maize) and one alfalfa cultivar (a closely-related plant to sainfoin).

## Materials and Methods

### Nematode Material

An original population of *M. enterolobii* was obtained from the Mbombela area where it infected roots of tomato (*Solanum lycopersicon*). A pure population was derived from single egg masses, and used for the experiments after confirmation of its identity using the SCAR-PCR method (Rashidifard et al., 2019b). The nematode was reared on roots of seedlings of the nematode susceptible tomato cultivar of “Moneymaker” (Fourie et al., 2012) and kept in a glasshouse at an ambient temperature range of 19–28°C and a photoperiod of 14L:10D. The main reason why *M. enterolobii* was selected for this study was the concerns raised regarding the widespread occurrence and pathogenicity of this species on various crops in SSA (Collett et al., 2021). Nematode infected tomato plants were uprooted 30 days after egg mass inoculation, and processed for nematode extraction using an adapted NaOCl extraction method (Riekert, 1995). The total numbers of eggs and second-stage juveniles (J2) for inoculation purposes were counted in a water suspension using a De Grisse counting dish (De Grisse, 1963) and Nikon SMZ 1 500 dissection microscope. Aliquots of ±500 eggs and J2 per pot were used to inoculate the plant roots.

### Plant Material and Nematode Infection

This experiment was designed to evaluate the rotation of crops in a maize-based cultivation system with the inclusion of sainfoin as a cover crop. The seven treatments (sequences) consisted of the following: monoculture maize (maize/maize), soybean/maize, sainfoin (Visnovsky)/maize, sainfoin (Perly)/maize, sainfoin (Taja)/maize, sainfoin (Esparsette)/maize, alfalfa/maize, and tomato/tomato (positive control). Seeds of these cultivars were obtained from the respective owner companies. The effect of each plant rotation in terms of the reduction of the population densities of *M*. *enterolobii* was compared to the treatment of monoculture maize.

One week before the experiment commenced, 0.5-L capacity, white plastic pots were filled with a sandy loamy soil [5.3% clay, 93.6% sand, 1.1% silt, 0.47% organic matter; pH (H_2_O) of 7.47)] that has been fumigated with Telone II (a.s. 1,3 dichloropropene @ a dosage rate of 120 l/ha) on June 16, 2020 and was kept under plastic cover in the sun for 3 weeks until 07 July when the first experiment started. The second experiment was conducted on October 07, 2020 using the same batch of soil. For all treatments, pots were sown with four seeds of the respective crop cultivars and maintained in a glasshouse at the temperature range of 20–28 ± 1.6°C and a photoperiod of 14L:10D. Two weeks after germination when the seedlings were at the two-leave stage, they were thinned to contain one seedling per pot and each plant root system was inoculated with ± 500 eggs and J2 of the *in vivo* reared *M. enterolobii* population (1 egg/J2 per cc) as this was reported as damage threshold for *Meloidogyne* (Bowen et al., 2008; Tiwari et al., 2019). Five weeks after nematode inoculation –required time for *M. enterolobii* to have at least one generation (Collett, 2020), all plants were uprooted, the aerial parts and roots cut off into small pieces (2 cm) and incorporated into the soil (4–8 cm deep). One week after the incorporation of the roots and aerial plant parts, all pots were sown with one maize seed, except for those designated to contain tomato, to which seedlings of Moneymaker were replanted. The pots were maintained in the greenhouse for 5 weeks under the same conditions described above for the preceding trial. The plants were watered four times a week or as necessary and each plant was also provided with 50 ml (2 g/L) Starke Ayres Nutrifeed (nitrogen 6.5%; phosphorous 2.7%; potassium 13.0%; calcium 7.0%; magnesium 2.2%; sulfur 7.5%) every second week.

At termination of the experiments, maize plants were removed from the pots and the areal parts discarded after measuring shoot lengths. The root system of each plant was weighed and then rinsed with running tap water. Eggs and J2 of *M. enterolobii* were extracted using the adapted NaOCl method of Riekert (1995) and counted as explained above. The reproduction factor (Rf) [final population (Pf) / initial population (Pi)] of the nematode population was determined for each treatment according to Oostenbrink’s reproduction factor (Windham and Williams, 1987). The experiment was repeated once (at a different time interval) using the same protocols (for nematodes, plant material, inoculation, maintenance) and glasshouse conditions as described above for the first study.

### Data Analysis

A randomized complete block design (RCBD) with six replicates (one plant per pot; six pots for each treatment) was chosen for the experimental layout for both experiments. Nematode data (eggs and J2 numbers per root system), were log(x) transformed, this also was applied for plant height in the repeat experiment. Data from each experiment was first subjected to an ANOVA (Statistica, Version 13.3) individually. Subsequently, Tukey’s HSD Test (*P* ≤ 0.05) was conducted to separate the means of the eight treatments of the two experiments. Combined data of both experiments was then subjected to a Factorial Analysis of Variance (ANOVA) (Statistica, Version 13.3) with time (representing the two experiments) as the main factor and treatments as the sub-factor. The effect of different treatments on control of nematode was calculated based on the following formula:

$$\left(\frac{X\times 100}{Y}\right)-100$$

where “X” is the final number of eggs and J2 and “Y” is the initial nematode population (*ca*. 500 eggs and J2). The graphs showing the results of both experiments independently were generated using GraphPad Prism version 8.02^1^.

## Results

Plant height, as well as the number of nematode eggs and J2 and the Rf values per root system of each crop cultivar were significantly different (*P* ≤ 0.05) among the treatments for both experiments. There were no significant differences for the root mass among the treatments of each experiment. Significant interaction was observed for all parameters for experiment x treatments, showing that the maize plants (treatments) reacted differently with regard to the *M. enterolobii* population densities representing the different crop treatments during the two experiments.

### Numbers of Eggs and J2s per Plant

A significant interaction (*P* = 0.000; *F* = 21.9) was observed for the numbers of eggs and J2 of *M. enterolobii* per root system for experiment × treatments (Supplementary Table 1) being significantly different (*P* ≤ 0.05) for the sainfoin (Taja)/maize rotation and the tomato standard for the two experiments.

**FIGURE 1:**
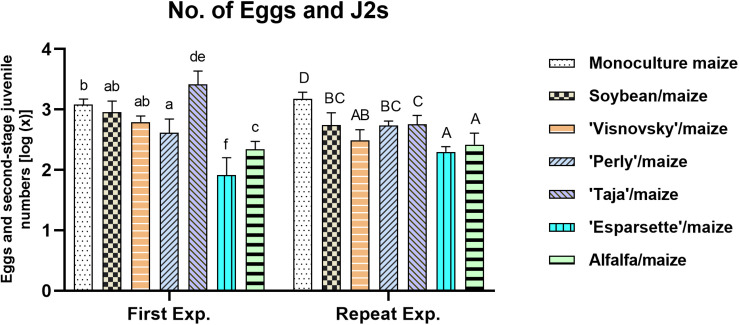
Population densities of *Meloidogyne enterolobii* in roots of maize, 12 weeks post-inoculation with ± 500 eggs and second-stage juveniles and incorporation of aerial material of previously grown maize (P-2432-R), soybean (DM-5953-RSF), sainfoin (Esparsette, Taja, Perly, Visnovsky), and alfalfa (BAR 7) into the soiland rotation thereof. The *y*-axis shows the log(x) transformed data. Different letters indicate significant differences at *P* ≤ 0.05 based on Tukey’s test for each experiment separately.

The rotation of sainfoin (Esparsette)/maize and soil amendment with the roots and aerial parts of this cultivar resulted in the lowest numbers of *M. enterolobii* eggs and J2 per root system (91 and 202, respectively) in the first and repeat experiments. Among the treatments (excluding tomato), soil amendment and rotation of the sainfoin Taja/maize had the highest number (2581) of eggs and J2 in the first experiment, whilst the monoculture maize showed the highest number of eggs and J2 (1541) in the repeat experiment. In the first experiment two cultivars of sainfoin (Perly and Esparsette), and alfalfa had significantly (*P* ≤ 0.05) lower number of eggs and J2 compared to monoculture maize (Figure 1). However, in this experiment the results showed that soil amendment and rotation of sainfoin (Taja) with maize increased the nematode population significantly (*P* ≤ 0.05). No significant differences (*P* ≤ 0.05) were observed when soybean used for soil amendment and rotated with maize compared to monoculture maize in the first experiment. However, the repeat experiment indicated that rotation and amendment of all treatments resulted in significantly (*P* ≤ 0.05) lower number of eggs and J2 per root system compared to monoculture maize.

### Effect of Rotation on Nematode Population Densities

**FIGURE 2:**
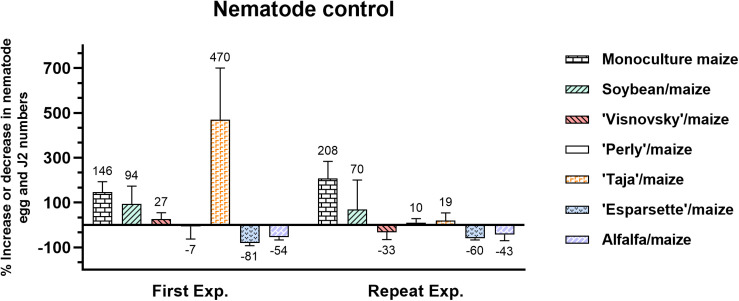
Variable *Meloidogyne enterolobii* density levels in roots of maize 12 weeks post-inoculation with ± 500 eggs and second-stage juveniles, and incorporation of aerial material of previously grown maize (P-2432-R), soybean (DM-5953-RSF), sainfoin (Esparsette, Taja, Perly, Visnovsky), and alfalfa (BAR 7) into the soil and rotation thereof of. The *y*-axis shows the percentage among which positive numbers show increase and negative numbers indicate reduction in nematode population.

Results from the first experiment showed that monoculture maize, soybean/maize and sainfoin (Visnovsky and Taja)/maize treatments increased *M. enterolobii* population densities, with Taja/maize having the highest (470%) and monoculture maize the second highest (146%) values (Figure 2). Sainfoin Perly, alfalfa and sainfoin Esparsette used for soil amendment and rotated with maize reduced nematode population densities by 7, 54, and 81%, respectively (Figure 2). In the repeat experiment monoculture maize had the highest increase (208%) in the *M. enterolobii* density, followed by soybean/maize (27%). Similar to the first experiment, soil amendment and rotation of sainfoin (Esparsette)/maize had the highest reduction (60%) in the nematode population density followed by the alfalfa/maize (43%). In contrast to the first experiment, sainfoin Taja/maize this time increased the nematode population by 19% (Figure 2).

### Rf Value

There was a significant interaction (*P* = 0.00; *F* = 156.8) for Rf value for experiment x treatment, which was the result of a significant difference (*P* ≤ 0.05) for tomato as the positive control in the first (8.7) and repeat (58.8) experiments (Supplementary Table 1).

**FIGURE 3:**
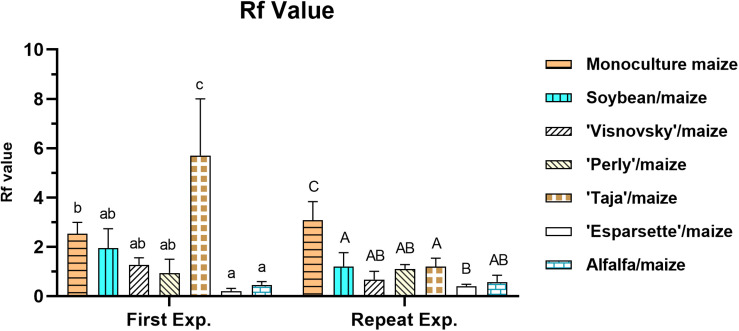
Reproduction factor (Rf) value of a *Meloidogyne enterolobii* population on maize, 12 weeks post-inoculation with ± 500 eggs and second-stage juveniles, and incorporation of aerial material of maize (P-2432-R), soybean (DM-5953-RSF), sainfoin (Esparsette, Taja, Perly, Visnovsky), and alfalfa (BAR 7) into the soil and rotation thereof. The *y*-axis shows Rf value. Different letters indicate significant differences at *P* ≤ 0.05 based on Tukey’s test for each experiment separately.

In the first experiment Rf values (excluding for tomato) ranged between 0.1 (sainfoin Esparsette/maize) and 2.4 (monoculture maize) with the former being significantly (*P* ≤ 0.05) lower, and sainfoin (Taja)/maize (5.7) significantly (*P* ≤ 0.05) higher than monoculture maize (Figure 3). In the repeat experiment Rf values ranged between 0.4 (sainfoin Esparsette/maize) and 3 (monoculture maize). In this experiment, all treatments had significantly (*P* ≤ 0.05) lower Rf values in comparison with monoculture maize (Figure 3).

### Plant Height

A significant interaction (*P* = 0.004; *F* = 3.53) for plant height for experiment x treatments was observed that can be ascribed by the significant difference (*P* ≤ 0.05) recorded for sainfoin (Taja)/maize 91.5 and 59 cm during the first and repeat experiments, respectively (Supplementary Table 2).

**FIGURE 4:**
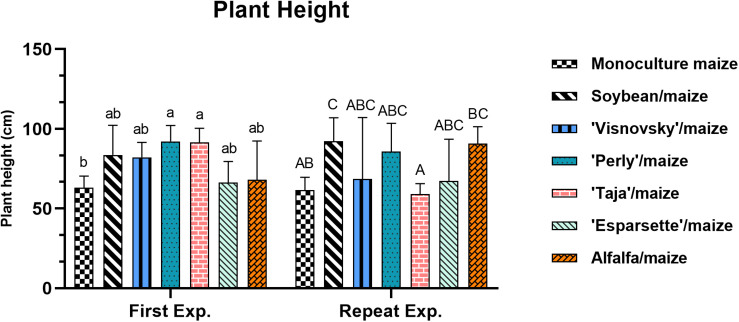
Plant height (cm) of maize plants, 12 weeks post-inoculation with ± 500 eggs and second-stage juveniles of *Meloidogyne enterolobii*, and incorporation of aerial material of maize (P-2432-R), soybean (DM-5953-RSF), sainfoin (Esparsette, Taja, Perly, Visnovsky), and alfalfa (BAR 7) into the soil and rotation thereof. The *y*-axis shows plant height value (cm). Different letters indicate significant differences at *P* ≤ 0.05 based on Tukey’s test for each experiment separately.

In the first experiment the plant height ranged from 63 cm (monoculture maize) to 92.1 cm (sainfoin Perly/maize). There were significant differences (*P* ≤ 0.05) between treatments in the first experiment, with soil amendment and rotation of sainfoin (Perly and Taja)/maize being significantly (*P* ≤ 0.05) taller than monoculture maize (Figure 4). Plant height values ranged between 56.1 cm (sainfoin Esparsette/maize) to 92.2 cm (soybean/maize) in the repeat experiment, with soybean/maize being significantly higher (*P* ≤ 0.05) than monoculture maize (Figure 4).

### Root Mass

**FIGURE 5:**
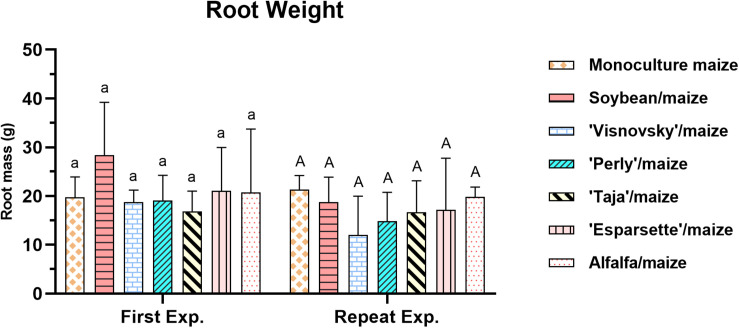
Root mass (g) of maize plants, 12 weeks post-inoculation with ± 500 eggs and second-stage juveniles of *Meloidogyne enterolobii*, and incorporation of aerial material of maize (P-2432-R), soybean (DM-5953-RSF), sainfoin (Esparsette, Taja, Perly, Visnovsky), and alfalfa (BAR 7) into the soil and rotation thereof. The *y*-axis shows plant weight (g). Different letters indicate significant differences at *P* ≤ 0.05 based on Tukey’s test for each experiment separately.

A significant interaction (*P* = 0.16; *F* = 6.045) for root weight for experiment × treatments was observed due to the significant differences (*P* ≤ 0.05) in root weight of the sainfoin: Taja/maize for the two experiments (Supplementary Table 2). No significant differences, however, existed among the treatments for the root weight in the first or repeat experiment (Supplementary Table 2). The root mass weight ranged between 16.8 g (sainfoin Taja/maize) to 21 g (soybean/maize and sainfoin (Esparsette)/maize) in the first experiment. In the repeat experiment, it ranged between 14.6 g (sainfoin Perly/maize) and 21.3 g (monoculture maize) and there was no significant difference (*P* ≤ 0.05) between different treatments (Figure 5).

## Discussion

In this study we observed significant reductions (compared to the initial/inoculated nematode population) of 81 and 60% of *M. enterolobii* population densities in the first and repeat experiments, respectively, when sainfoin cultivar Esparsette was rotated with maize and incorporated into the soil. This result highlights the positive role sainfoin can potentially play in grain-based sequences to minimize damage caused by *M. enterolobii*. Substantial reductions of 54 and 43% of *M. enterolobii* population densities (in the first and repeat experiments, respectively) when using the nematode resistant alfalfa cultivar BAR 7 in rotation with maize, further emphasizes the beneficial impact of using sainfoin for nematode control in cropping sequences. For sainfoin cultivar Taja contradicting results were observed with regard to the final *M. enterolobii* densities between the first and repeat experiment. Possible explanations that compromised these results could be that the inoculation effectivity in terms of numbers (although the same inoculation protocol was followed and the same *M. enterolobii* population was used) and/or hatching rate of J2 varied between the two experiments, and/or that potential differences in root growth of Taja existed in the respective experiments. These results, however, accentuated that growing Taja may lead to increased population densities of *M. enterolobii*. Similar experiences were recorded by Rashidifard et al. (2019a) and Agenbag (2016) in terms of differences recorded for root-knot final nematode population densities for initial and repeat experiments conducted with tomato.

The significant inhibiting effects of sainfoin cultivar Esparsette on *M. enterolobii* population densities suggests that it can be a potential alternative for alfalfa cultivar BAR 7 (reported as resistant to pest and disease)^2^ in areas where alfalfa pests, such as *Sitona* weevils, are a serious concern. In addition, it is important to take other advantages of sainfoin into consideration. Sainfoin was reported to be resistant to various pests that feed on alfalfa (Wallace, 1969; Lance, 1980). The condensed tannin present in sainfoin were reported to have non-bloating and anthelmintic effects (Novobilský et al., 2013).

The significantly lower numbers of *M. enterolobii* eggs and J2s on maize rotated with sainfoin cultivars Perly and Esparsette, and alfalfa (BAR 7) in the first experiment (compared to monoculture maize) opposed to similar population densities on maize rotated with soybean, demonstrate the potential use of these fodders as an alternative in traditional grain-based cropping systems. The high susceptibility of soybean to the same nematode pests that parasitize maize poses problems in SSA (Fourie et al., 2017) and other regions (Coyne et al., 2018; Dababat and Fourie, 2018) and could compromise the sustainable production of grain crops (Riekert and Henshaw, 1998; Miller et al., 2006).

The low Rf data obtained in both experiments, revealed that sequences with sainfoin cultivar Esparsette and alfalfa cultivar BAR 7 may be resistant to *M. enterolobii*. This should, however, be verified in host status experiments since this study was only investigating cropping sequences of the selected crops.

The current study showed high variation among sainfoin cultivars in terms of their effectiveness in reducing root-knot nematode population density levels when used in a crop rotation system. Additionally, the inhibiting effects of sainfoin on *M. enterolobii* recorded in our study were in contrast with previous reports that showed high susceptibility of sainfoin to *M. hapla* (Griffin, 1971; Wofford and Gray, 1987; Shigaki et al., 2008). This could be attributed to the high genetic diversity that was reported among different sainfoin cultivars (Mohajer et al., 2013; Zarrabian et al., 2013). It is speculated that the incorporation of leaves of sainfoin into soil substrate would result in the release of condensed tannins. The released tannins might then play a role in suppression of *M. enterolobii* in soil or in the plant tissues where this nematode pest feeds. Further studies will be needed to analyze the impact of condensed tannins in control of PPNs.

The value of a crop rotation and soil amendment with soybean, sainfoin and alfalfa reflected by maize height data was greater than monoculture maize in both experiments. This is a common phenomenon when crop rotation with legumes is practiced and it is in agreement with previous reports (Tanimu et al., 2007). High numbers of *M. enterolobii* population densities did not show a negative effect on plant height, showing that this cannot be used as an indication for nematode damage in these kind of experiments.

The root mass value for both experiments showed no significant differences among the treatments which is agreed with the results reported from a local study (Rashidifard et al., 2019a) and warrants no further discussion.

Besides this study, there has been only one report regarding a tolerant cultivar of sainfoin (Gray et al., 2006a), “Shoshone,” identified as tolerant to *M. hapla*. Other sainfoin cultivars that have been tested for their host status against *M. hapla* and *Ditylenchus destructor*, showed high susceptibility to these nematodes (Wofford and Gray, 1987; Wofford et al., 1989; Shigaki et al., 2008). Reporting the beneficial effects of sainfoin cultivar Esparsette, which is the only available cultivar in South Africa, is a novel addition to the limited, existing research on this crop. The fact that this cultivar was tested against *M. enterolobii*, known as an emerging threat for agriculture production and food security in SSA (Coyne et al., 2018; Visagie et al., 2018; Rashidifard et al., 2019b; Bello et al., 2020; Collett et al., 2021) could be an advantage for producers and crop industries.

## Conclusion

In this paper, we showed that introducing sainfoin in a crop rotation system is a novel nematode management strategy with potential to be used by producers across the globe. Especially, sainfoin cultivar Esparsette showed a high efficacy in nematode reduction, making it a promising candidate to be used in rotation with maize and as cover crop for soil amendment in SSA where PPNs are a serious concern. More studies need to be conducted to elucidate the host status of different sainfoin cultivars against *Meloidogyne* and other economically important PPNs such as *Pratylenchus* species, with the role that condensed tannins play in this regard to be emphasized.

## Data Availability Statement

The original contributions presented in the study are included in the article/Supplementary Material, further inquiries can be directed to the corresponding author/s.

## Author Contributions

MR and SA developed the project and designed the experiments and drafted the manuscript. MR executed the experiments, collected, analyzed, and interpreted the data. SC, TT, and HF contributed to the design of the work. All authors reviewed and edited the manuscript.

## Conflict of Interest

The authors declare that the research was conducted in the absence of any commercial or financial relationships that could be construed as a potential conflict of interest.

## Footnotes

1 http://www.graphpad.com

2 https://www.barenbrug.co.za/forage/products/lucerne/bar-7.htm
