# Supplementary material for: A Pilot Approach Investigating the Potential of Crop Rotation With Sainfoin to Reduce Meloidogyne enterolobii Infection of Maize Under Greenhouse Conditions
Source: Front Plant Sci. 2021 Apr 16;12:659322. doi: 10.3389/fpls.2021.659322 (PMC8086598; doi:10.3389/fpls.2021.659322)
Supplement: Supplementary file 1 [file Data_Sheet_1.docx]

**Supplementary table 1.** *Meloidogyne enterolobii* reproduction data determined for genotypes of maize, sainfoin, soybean and tomato of which seedlings were inoculated with ±500 eggs and second-stage juveniles (J2) and kept in a glasshouse for 12 weeks.

| **Treatments** | **Numbers of eggs and J2/root system** | | **Rf Value^1^** | |
| --- | --- | --- | --- | --- |
|  | First Exp. | Repeat Exp. | First Exp. | Repeat Exp. |
| **Maize (‘P-2432-R’) / Maize** | 3.1^2^ (1229^3^ ± 236^4^) bd EFG | 3.1 (1541 ± 375) d FG | 2.4 b AB | 3 c AB |
| **Soybean (‘DM-5953-RSF’) / Maize** | 2.9 (972 ± 393) ab EF | 2.7 (603 ± 277) bc CDE | 1.9 ab AB | 1.2 a AB |
| **Sainfoin (‘Visnovsky’) / Maize** | 2.7 (634 ± 140) ab DE | 2.4 (335 ± 164) ab BCD | 1.2 ab AB | 0.6 ab A |
| **Sainfoin (‘Perly’)/ Maize** | 2.6 (467 ± 280) ac BCD | 2.7 (551 ± 89) bc CDE | 0.9 ab AB | 1.1 ab AB |
| **Sainfoin (‘Taja’) / Maize** | 3.4 (2851 ± 1147) de GH | 2.7 (595 ± 172) c DE | 5.7 c BC | 1.1 a AB |
| **Sainfoin (‘Esparsette’) / Maize** | 1.9 (91 ± 60) f A | 2.2 (202 ± 38) a B | 0.1 a A | 0.4 b A |
| **Alfalfa (‘BAR 7’) / Maize** | 2.3 (228 ± 63) c B | 2.4 (284 ± 137) a BC | 0.4 ab A | 0.6 ab A |
| **Tomato (‘Moneymaker’)** | 3.6 (4393 ± 1013) e H | 4.4 (29448 ± 4387) e I | 8.7 d C | 58.8 d D |
| ***P* value** | 0.00 | 0.00 | 0.000 | 0.00 |
| ***F* value** | 51.89 | 150.2 | 37.91 | 249.1 |
| **Interaction data: Experiments x treatments** | | | | |
| ***P* value** | 0.000 | | 0.00 | |
| ***F* value** | 21.9 | | 156.8 | |

^1^ Rf = final egg and J2 numbers (*P*f)*/*initial egg and J2 numbers (*P*i) (Windham & Williams, 1987).

^2^ Log (x) transformed value.

^3^ Real means.

^4^ Standard Deviation from the real means (Tukey’s Test at P ≤ 0.05); Lower case letters indicate differences in egg and J2 numbers and reproduction parameters among treatments for each individual experiment, with means in each column followed by the same letter not differing significantly at P ≤ 0.05; upper case letters indicate differences in egg and J2 numbers and reproduction parameters among the similar treatments between the two experiments, with means in each line followed by the same letter not differing significantly at P ≤ 0.05

Supplementary **table 2.** Plant height (cm) and root mass (g) determined for genotypes of alfalfa, maize, sainfoin, soybean of which inoculated with 500 eggs and second-stage juveniles (J2) of *Meloidogyne enterolobii* and kept in a glasshouse for 12 weeks.

| **Treatments** | Plant height | | Root mass | |
| --- | --- | --- | --- | --- |
|  | First Exp. | Repeat Exp. | First Exp. | Repeat Exp. |
| **Maize (‘P-2432-R’) / Maize** | 63^1^ ± 7.3^2^ b AB | 61.6 ± 7.9 ab AB | 19.7 ± 4.1 a AB | 21.3 ± 2.8 a AB |
| **Soybean (‘DM-5953-RSF’) / Maize** | 83.6 ± 18.5 ab ABC | 92.2 ± 14.7 c BC | 21 ± 10.8 a B | 18.7 ± 5.1 a AB |
| **Sainfoin (‘Visnovsky’) / Maize** | 82.1 ± 9.2 ab ABC | 68.5 ± 21.3 abc ABC | 18.7± 2.4 a AB | 12 ± 6.9 a A |
| **Sainfoin (‘Perly’) / Maize** | 92.1 ± 9.9 a C | 57.1 ± 17.6 abc ABC | 19 ± 5.1 a AB | 14.8 ± 5.8 a AB |
| **Sainfoin (‘Taja’) / Maize** | 91.5 ± 8.8 a BC | 59 ± 6.5 a A | 16.8 ± 4.1 a AB | 16.6 ± 6.4 a AB |
| **Sainfoin (‘Esparsette’) / Maize** | 66.3 ± 13.2 ab ABC | 56.1 ± 20 abc ABC | 21 ± 8.8a AB | 17.1 ± 8.3 a AB |
| **Alfalfa (‘BAR 7’) / Maize** | 68 ± 24.3 ab ABC | 75.6 ± 10.5 bc ABC | 20.7 ± 12.9 a AB | 19.8 ± 1.8 a AB |
| **Tomato (‘Moneymaker’)** | - | - | 20.4 ± 2.9 a AB | 15.6 ± 6.2 a AB |
| ***P* value** | 0.003 | 0.02 | 0.317 | 0.316 |
| ***F* value** | 4.097 | 4.581 | 1.218 | 1.219 |
| **Interaction data: Experiments x treatments** | | | | |
| ***P* value** | 0.004 | | 0.016 | |
| ***F* value** | 3.53 | | 6.045 | |

^1^ Real means.

^2^ Standard Deviation from the real means (Tukey’s Test at P ≤ 0.05); Lower case letters indicate differences in plant parameters among treatments for each individual experiment, with means in each column followed by the same letter not differing significantly at P ≤ 0.05; upper case letters indicate differences in plant parameters among treatments between the two experiments, with means in each line followed by the same letter not differing significantly at P ≤ 0.05
